# Intranasal insulin rescues repeated anesthesia-induced deficits in synaptic plasticity and memory and prevents apoptosis in neonatal mice via mTORC1

**DOI:** 10.1038/s41598-021-94849-3

**Published:** 2021-07-29

**Authors:** Patricia Soriano Roque, Mehdi Hooshmandi, Laura Neagu-Lund, Shelly Yin, Noosha Yousefpour, Hiroaki Sato, Tamaki Sato, Yosuke Nakadate, Akiko Kawakami, Soroush Tahmasebi, Alfredo Ribeiro-da-Silva, Christos G. Gkogkas, Masha Prager-Khoutorsky, Thomas Schricker, Linda Wykes, Arkady Khoutorsky

**Affiliations:** 1 Department of Anesthesia and Faculty of Dentistry, McGill University, Montreal, QC Canada; 2 School of Human Nutrition, McGill University, Montreal, QC Canada; 3 Department of Pharmacology and Therapeutics, McGill University, Montreal, QC Canada; 4 Department of Anesthesia, McGill University Health Centre Glen Site, Royal Victoria Hospital, Montreal, QC Canada; 5 Department of Pharmacology and Regenerative Medicine, University of Illinois at Chicago, Chicago, IL 60612 USA; 6 Alan Edwards Centre for Research on Pain, McGill University, Montreal, QC Canada; 7 Department of Anatomy and Cell Biology, McGill University, Montreal, QC Canada; 8 Division of Biomedical Research, Institute of Molecular Biology and Biotechnology, Foundation for Research and Technology-Hellas, University Campus, 45110 Ioannina, Greece; 9 Department of Physiology, McGill University, Montreal, QC H3G 1Y6 Canada

**Keywords:** Neuroscience, Mechanisms of disease

## Abstract

Long-lasting cognitive impairment in juveniles undergoing repeated general anesthesia has been observed in numerous preclinical and clinical studies, yet, the underlying mechanisms remain unknown and no preventive treatment is available. We found that daily intranasal insulin administration to juvenile mice for 7 days prior to repeated isoflurane anesthesia rescues deficits in hippocampus-dependent memory and synaptic plasticity in adulthood. Moreover, intranasal insulin prevented anesthesia-induced apoptosis of hippocampal cells, which is thought to underlie cognitive impairment. Inhibition of the mechanistic target of rapamycin complex 1 (mTORC1), a major intracellular effector of insulin receptor, blocked the beneficial effects of intranasal insulin on anesthesia-induced apoptosis. Consistent with this finding, mice lacking mTORC1 downstream translational repressor 4E-BP2 showed no induction of repeated anesthesia-induced apoptosis. Our study demonstrates that intranasal insulin prevents general anesthesia-induced apoptosis of hippocampal cells, and deficits in synaptic plasticity and memory, and suggests that the rescue effect is mediated via mTORC1/4E-BP2 signaling.

## Introduction

Repeated general anesthesia during the early postnatal period causes neurotoxicity and long-lasting cognitive deficits in a wide range of animal species^1–3^. Although short anesthetic exposure in children does not lead to cognitive impairment^4^, animal studies and pediatric epidemiological reports linked repeated or prolonged general anesthesia to cognitive and behavioral abnormalities, including neurodevelopmental delay, learning disabilities, and attention deficit/hyperactivity disorder^5–7^. The accumulating evidence in animal and human studies prompted the U.S. FDA Drug Safety Communication to issue an alert on the pediatric use of anesthetic and sedative agents, warning that they may negatively impact brain development upon repeated or prolonged administration to young children (or the fetus during the third trimester of pregnancy)^8^. The mechanisms of repeated anesthesia-induced cognitive deficits remain elusive and no treatment to prevent memory deficits is available. In juvenile animals, repeated general anesthesia induces cell death in the brain via apoptosis^2^, which is a proposed mechanism underlying persistent dysfunction of neuronal circuits, impaired synaptic plasticity, and long-term memory formation in adulthood.

Intranasal insulin allows a non-invasive and efficient delivery of insulin into the brain. This approach has been recently tested and found effective in several studies as a potential treatment for cognitive deficits in Alzheimer’s disease, brain injury and other brain disorders^9–13^. Previous studies have shown that extremes of age are vulnerable to prolonged anesthesia exposure and demonstrated that aged mice exhibit repeated anesthesia-induced cognitive impairment that can be alleviated by intranasal insulin^14^.

The hippocampus is highly vulnerable to neurotoxicity^15^ and is one of the first regions where cells undergo apoptosis upon general anesthesia in juvenile animals^16^. It has central roles in acquisition and consolidation of different types of memory, particularly spatial and contextual memory^17^, therefore, an early life damage to the hippocampus results in long-lasting memory impairments^18^.

Insulin receptors are widely expressed in the mammalian brain and are enriched in the hippocampus^19^. Binding of insulin to the insulin receptor activates several signaling pathways including the mechanistic target of rapamycin complex 1 (mTORC1). mTORC1 is a master regulator of mRNA translation, which is also involved in regulation of other processes such as lipid biogenesis, and autophagy^20^. mTORC1 regulates mRNA translation via phosphorylation-mediated inhibition of its major downstream effector and translational repressor, eukaryotic translation initiation factor 4E (eIF4E)-binding protein (4E-BP) and ribosomal protein S6 kinase (S6K)^20,21^. mTORC1 promotes mitochondrial functions and energy metabolism via preferential 4E-BP-dependent translation of mitochondria-related mRNAs^22^. Mitochondrial dysfunction, caused by repeated general anesthesia in juvenile animals, critically contributes to the induction of apoptosis and subsequent long-lasting cognitive deficits^23,24^. We, therefore, hypothesized that activation of the mTORC1/4E-BP2 pathway by insulin may have beneficial effects on the brain by enhancing mitochondrial functions, and thereby rendering hippocampal cells more resistant to anesthesia-induced cellular stress.

Herein, we demonstrate that daily administration of intranasal insulin for 7 days before repeated general anesthesia of juvenile mice prevents anesthesia-induced apoptosis in the hippocampus and rescues deficits in synaptic plasticity and hippocampus-dependent memory in adulthood. Furthermore, we show that intranasal insulin prevents apoptosis of hippocampal cells by activating the mTORC1 pathway, since the beneficial effect of insulin on apoptosis was abolished by the mTORC1 inhibitor, temsirolimus. Collectively, our results demonstrate that intranasal insulin prevents anesthesia-induced apoptosis in juvenile mice by activating the mTORC1 pathway, and reverses deficits in synaptic plasticity and memory in adulthood.

## Results

### Administration of intranasal insulin prior to anesthesia exposure in juvenile mice prevents memory impairment in adulthood

We first studied whether intranasal insulin administered prior to repeated anesthesia exposure in young mice rescues the subsequent memory deficits in adulthood. In clinical practice, children undergo prolonged and recurrent surgical procedures and thus are exposed to repeated anesthesia^25^. To model this situation, we subjected postnatal day (PD) 15 mice to clinically relevant 1.5% isoflurane anesthesia for two hours a day over three consecutive days, for a total of six hours. Isoflurane and its derivative sevoflurane are commonly used for anesthesia maintenance, especially for prolonged, over one-hour procedures. Mice were exposed to anesthesia on postnatal days 15–17, which correlate to ~ 2-year-old children based on weaning age and lifespan^26^, to avoid metabolic derangement and hypercarbia, which are often observed during anesthesia in younger (e.g. PD 7) rodents^27^.

**Figure 1:**
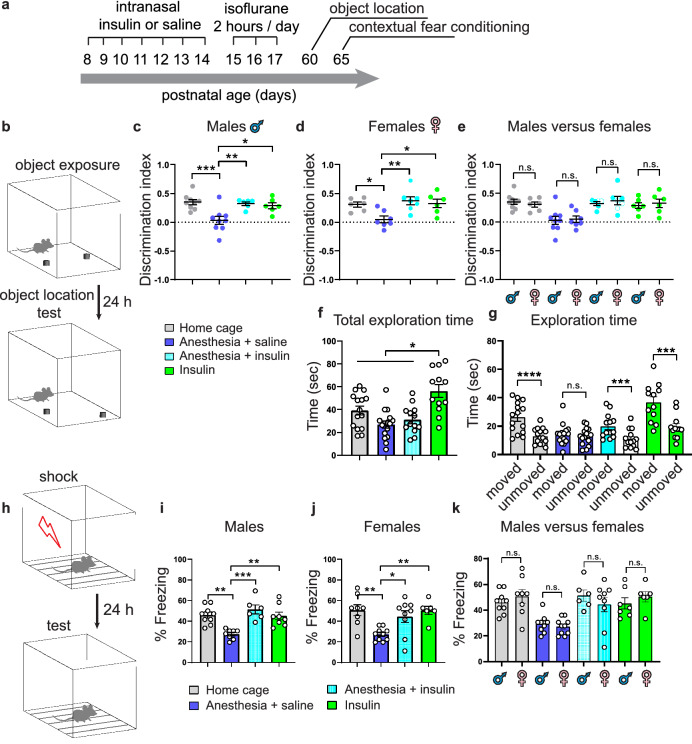
Pre-treatment with intranasal insulin prevents repeated general anesthesia-induced impairment in memory formation in male and female mice. (**a**) Diagram depicting experimental design. Mice were pre-treated daily (postnatal days 8–14) with intranasal insulin or saline before repeated general anesthesia (1.5% isoflurane, 2 h/day) at postnatal days 15–17. (**b**) Schematic representation of a novel object location test. Male (**c**) and female (**d**) adult mice, subjected to repeated general anesthesia postnatally, exhibit decreased discrimination index (time exploring moved object minus time exploring unmoved object divided by total time exploring both objects) and the decrease is prevented by pre-treatment with intranasal insulin (Home cage: n = 9 males and 6 females; Anesthesia + saline: n = 9 males and 7 females; Anesthesia + insulin: n = 6 males and 7 females; Insulin: n = 6 males and 6 females, one-way ANOVA followed by Tukey’s multiple comparisons post hoc test). (**e**) Comparison between males and females shows no sex-specific effects. Total exploration time (both sexes combined) (**f**) and exploration of moved and unmoved objects (**g**) are shown. (**h**) Schematic representation of a contextual fear conditioning test. Long-term fear memory (24 h after training, recorded during 5 min period) is impaired in anesthesia-exposed mice in males (**i**) and females (**j**), and this deficit is prevented by pre-treatment with intranasal insulin (Home cage: n = 9 males and 8 females; Anesthesia + saline: n = 8 males and 10 females; Anesthesia + insulin: n = 6 males and 9 females; Insulin: n = 8 males and 6 females, one-way ANOVA followed by Tukey’s multiple comparisons post hoc test). (**k**) comparison between males and females. Data are presented as mean ± SEM. n.s. (not significant), *p < 0.05, **p < 0.01, ***p < 0.001, ****p < 0.0001.

Following repeated anesthesia at PD 15–17, mice were allowed to mature and hippocampal-dependent memory was assessed in adult animals (PD 60–65) using object location and contextual fear conditioning tests (Fig. 1a). Whereas control male (Fig. 1b,c) and female (Fig. 1d) mice spent significantly more time exploring the moved object in the novel object location test, anesthesia-exposed mice did not discriminate between the two objects, indicating an impairment of spatial memory in mice subjected to anesthesia. Intranasal insulin was administered starting at PD 8 for seven consecutive days (2 units daily) prior to repeated general anesthesia at PD 15–17 (Fig. 1a). The seven day-regimen of intranasal insulin administration was adapted from previous studies in adult^14,28^ and juvenile mice^29,30^. Remarkably, male (Fig. 1c) and female (Fig. 1d) mice treated with intranasal insulin before the anesthesia exhibited no memory deficits. This indicates that intranasal insulin prior to anesthesia protects against anesthesia-induced memory impairment in adulthood. Administration of intranasal insulin alone in juvenile mice had no effect on memory in adulthood (Fig. 1c,d). Comparison between male and female animals showed no sex-specific differences (Fig. 1e). Total exploration time remained unaltered in anesthesia and anesthesia with insulin groups, however, it was increased in insulin alone group (Fig. 1f,g).

In the contextual fear conditioning test, anesthesia-exposed male (Fig. 1h,i) and female (Fig. 1j) mice exhibited significantly reduced freezing behavior as compared to control animals 24 h after the training, indicating an impairment of long-term contextual memory in both sexes. Similar to the results obtained in the novel object location test, mice treated with intranasal insulin prior to repeated general anesthesia showed no memory deficits (males, Fig. 1i; females, Fig. 1j). Comparison between male and female mice showed no sex-specific effects (Fig. 1k). Collectively, these results indicate that the treatment of juvenile mice with intranasal insulin prior to repeated general anesthesia prevents long-lasting hippocampal-dependent memory deficits in adulthood in both sexes.

**Figure 2:**
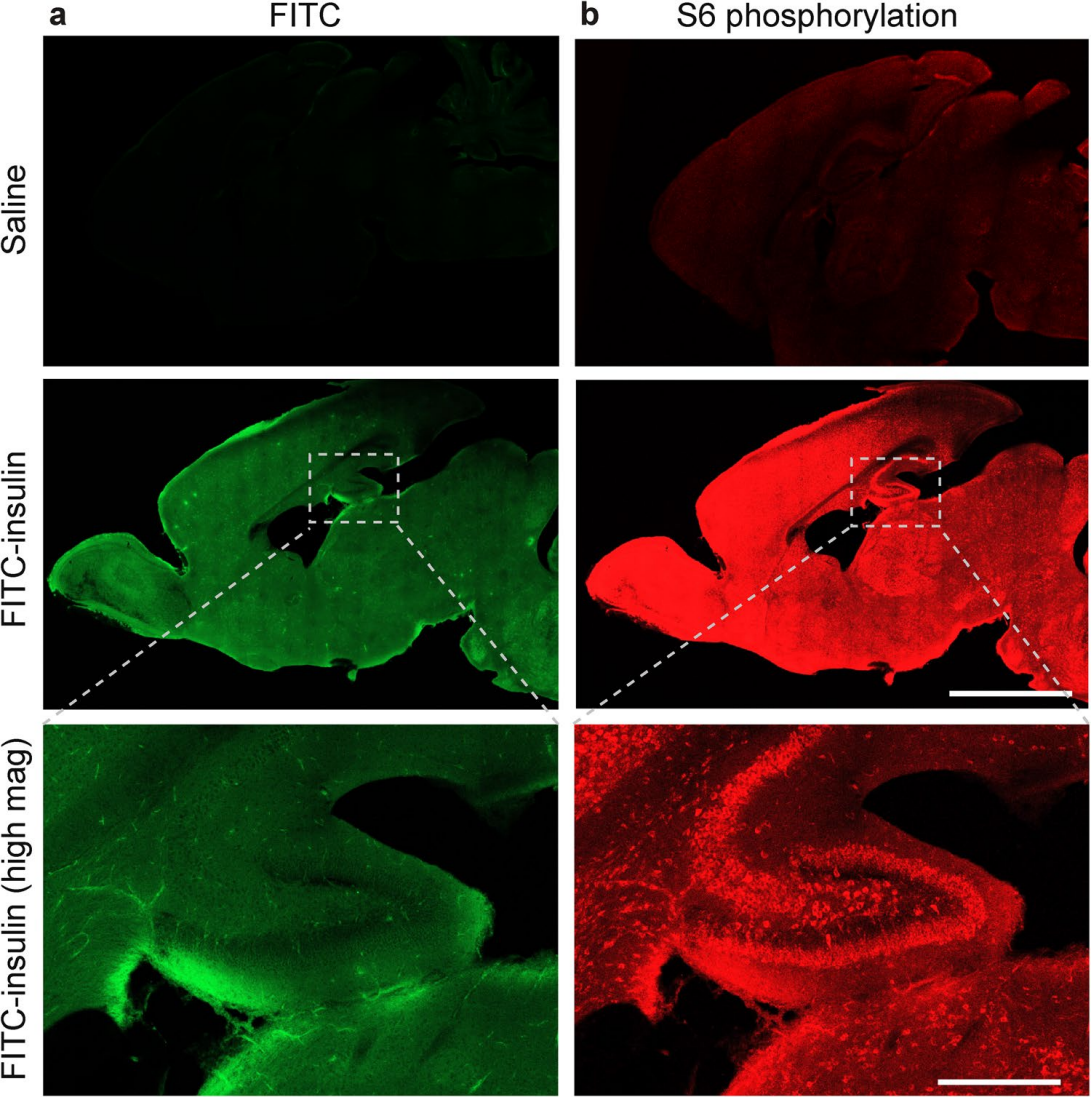
Insulin applied intranasally rapidly penetrates the brain and strongly activates mTORC1 activity. (**a**) Postnatal day 8 mice were treated with intranasal FITC-insulin or saline and their brains were fixed 30 min post-administration and immunostained with phospho-S6-specific antibody. Imaging of FITC fluorescence reveals widespread FITC distribution in the brain, indicating the presence of the FITC-insulin in the brain (left). (**b**) FITC-insulin activates mTORC1 signaling as evidenced by increased phosphorylation of mTORC1 downstream effector, S6. Bottom images represent magnification of the grey rectangle-marked area in the middle images. Scale bar (middle) 4 mm, (bottom) 700 µm.

To confirm that insulin applied intranasally reaches the hippocampus, we delivered FITC-labeled insulin intranasally in PD 8 pups and fixed the brain 30 min post-insulin administration. Imaging of brain sections showed a widespread distribution of fluorescent FITC signal in the brain (Fig. 2a). To confirm that the insulin could stimulate intracellular signaling cascades, we performed immunostaining for phosphorylated S6 (p-S6). P-S6 is a downstream effector and the most reliable readout of mTORC1 pathway, which is activated by binding of insulin to the insulin receptor^20^. We detected increased levels of p-S6 in hippocampal neurons 30 min after intranasal insulin administration (Fig. 2b). Altogether, these experiments demonstrate that intranasally administered insulin rapidly penetrates the brain and activates mTORC1 in hippocampal neurons and other brain areas.

### Pretreatment with intranasal insulin rescues anesthesia-induced deficits in synaptic plasticity

**Figure 3:**
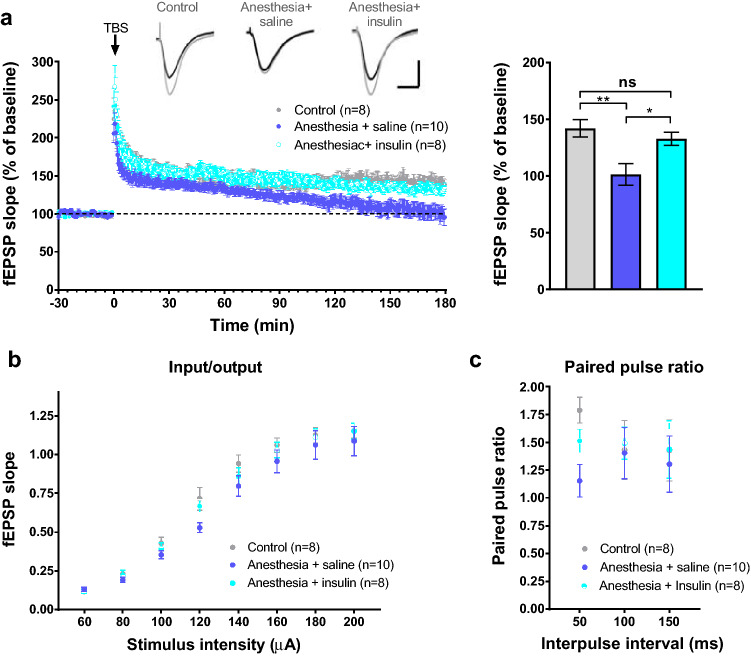
Intranasal insulin prevents repeated anesthesia-induced impairment in L-LTP. (**a**) Acute hippocampal slices were prepared from adult mice and field excitatory postsynaptic potentials (fEPSPs) were recorded in the CA1 hippocampal area following theta-burst stimulation (TBS) of the Schaffer collateral pathway. Representative traces at 3-h time-point are shown for each condition (baseline in black and 3 h post-TBS in grey). Column bars on the right show quantification of fEPSP slope (% of baseline) during the last 10 min of recording (average responses during 170–180 min post-TBS). Whereas the late-phase of long-term potentiation (L-LTP, 3 h after stimulation) is impaired in anesthesia-exposed mice, this impairment is rescued in mice pretreated with intranasal insulin (*F*_2, 23_ = 7.204; control (n = 8 slices from 8 mice) versus Anesthesia + saline (n = 10 slices from 10 mice), **p < 0.01; Anesthesia + saline versus Anesthesia + insulin (n = 8 slices from 8 mice), *p < 0.05; one-way ANOVA followed by Tukey’s multiple comparisons post hoc test). (**b**) No differences were found between the three experimental groups in input–output curves over a wide range of stimulus intensities. (**c**) Paired-pulse facilitation is not different between groups. For statistical analysis in (**b**) and (**c**), two-way ANOVA followed by Tukey’s multiple comparisons post hoc test was used. Data are presented as mean ± SEM.

A cellular neurophysiological correlate of learning and memory is long-term potentiation (LTP), which is one of the most characterized forms of synaptic plasticity^31^. It has been shown that the protein synthesis-dependent late phase of LTP (L-LTP) is impaired in adult animals that were exposed to repeated or prolonged general anesthesia during early postnatal period^32^. To study whether intranasal insulin pre-treatment prior to repeated general anesthesia in juvenile mice rescues the deficits in synaptic plasticity in adult animals, we measured L-LTP in acute hippocampal slices from adult (8 weeks of age) control mice as well as mice subjected to repeated general anesthesia which were pretreated with intranasal insulin or vehicle during early postnatal period. Stimulation of the Schaffer collateral pathway in stratum radiatum (CA1) with theta-burst stimulation (TBS) induced long-lasting potentiation of field excitatory postsynaptic potential (fEPSP) in control mice (Fig. 3a). L-LTP was impaired in mice subjected to repeated anesthesia (fEPSP slope normalized to baseline; control: 142.04 ± 7.64%; anesthesia + vehicle: 101.34 ± 9.52%; n = 8–10 mice/condition, p < 0.01, Fig. 3a). Interestingly, intranasal insulin-pretreated mice showed persistent synaptic potentiation which was comparable to the control group (control: 142.04 ± 7.64%; anesthesia + insulin: 132.8 ± 5.81%, p > 0.05, Fig. 3). We observed no alterations in input–output relationships (Fig. 3B) and the paired pulse facilitation (Fig. 3c) in any experimental group, indicating that basal synaptic transmission remained unchanged. These results indicate that intranasal insulin rescues repeated general anesthesia-induced impairment in L-LTP.

### Pre-treatment with intranasal insulin rescues anesthesia-induced apoptosis in juvenile mice via mTORC1

**Figure 4:**
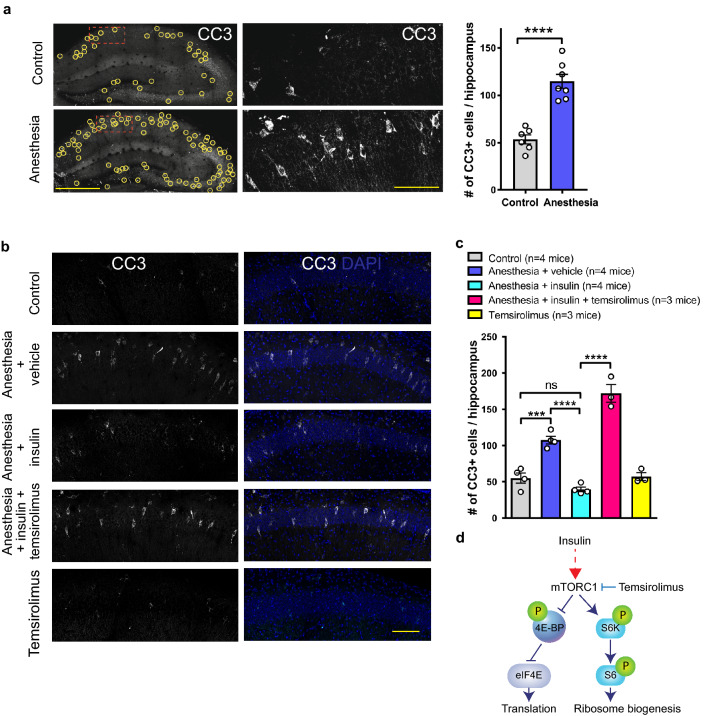
Intranasal insulin prevents the induction of apoptosis following repeated general anesthesia in juvenile mice via activation of mTORC1. (**a**) Repeated general anesthesia (1.5% isoflurane, 2 h/day) was performed on postnatal days 15–17 mice, and the brains were fixed one hour after the last anesthesia session at postnatal day 17 and dorsal hippocampus sections were immunostained against cleaved caspase 3 (CC3). Representative images of hippocampus showing CC3 at low (left) and high (middle) magnification. Yellow circles indicate the location of CC3-positive cells. (Right) Quantification of CC3-positve cells. The numbers of CC3-positive cells in three sequential dorsal hippocampus sections (separated by 200 µm, averaged for both hippocampi) was calculated per animal. Anesthesia increases the number of CC3-positive cells in the hippocampus (****p < 0.0001, a student’s t-test, control n = 6, anesthesia n = 7). (**b**) Repeated general anesthesia (1.5% isoflurane, 2 h/day, postnatal days 15–17) was performed on mice pre-treated daily during postnatal days 8–14 with saline, insulin, insulin and temsirolimus (applied ICV 20 min before intranasal insulin), or temsirolimus alone. The brains were fixed one hour after the last anesthesia session at postnatal day 17 and hippocampal sections were immunostained against cleaved caspase 3 (CC3). Representative images of CA1 hippocampal area showing CC3 (white) and DAPI (blue). **(c)** Quantification of CC3-positve cells. The increase in the number of CC3-positive cells following repeated general anesthesia is prevented by intranasal insulin (*F*_5, 15_ = 76.09; Control (n = 4) versus Anesthesia + vehicle (n = 4), ***p < 0.001; Anesthesia + vehicle versus Anesthesia + insulin (n = 4), ****p < 0.0001, one-way ANOVA followed by Tukey’s multiple comparisons post hoc test). Temsirolimus (2 µl at 50 µg/µl injected icv 20 min before intranasal insulin administration) prevents the rescue effect of intranasal insulin (Anesthesia + insulin (n = 4) versus Anesthesia + insulin + temsirolimus (n = 3), ****p < 0.0001). Data points represent individual mice. Data are presented as mean ± SEM. **(d)** Diagram showing the activation of mTORC1 by insulin and its inhibition by temsirolimus. 4E-BPs and S6K are two major downstream effectors of mTORC1.

General anesthesia-induced apoptosis in juvenile mice is a proposed mechanism underlying persistent memory deficits. To determine whether intranasal insulin rescues synaptic plasticity and memory deficits in the hippocampus by preventing apoptosis following repeated general anesthesia, we assessed apoptosis using immunostaining for a commonly used apoptotic marker cleaved caspase 3 (CC3)^33^. As expected, three consecutive days of anesthesia (1.5% isoflurane, 2 h a day, PD 15–17) induced a large increase in the number of apoptotic cells in the hippocampus one hour after the last anesthesia session (an increase of 114% from 53.5 ± 4.86 cells in control mice to 114.6 ± 7.42 cells in anesthesia group, p < 0.0001, Fig. 4a).

Intranasal insulin administration prior to repeated general anesthesia exposure prevented the induction of apoptosis in the hippocampus as the number of CC3-positive cells was not significantly different from controls (control: 55.13 ± 6.99 cells; anesthesia + insulin: 39.5 ± 3.3 cells, p > 0.05, Fig. 4b,c).

mTORC1 is the main intracellular signaling pathway activated by binding of insulin to its receptor (Fig. 4d). To test whether mTORC1 activation mediates the beneficial effects of insulin on the anesthesia-induced apoptosis, we delivered a specific mTORC1 inhibitor temsirolimus, using intracerebroventricular (icv) administration, 20 min prior to the intranasal insulin treatment^34,35^. Control mice received vehicle (icv). Blocking mTORC1 in the brain prevented the anti-apoptotic effect of intranasal insulin during anesthesia exposure, as mice given temsirolimus prior to intranasal insulin showed significantly more CC3-positive cells after anesthesia as compared to insulin pre-treated animals (anesthesia + insulin: 39.5 ± 3.3 cells; anesthesia + insulin + temsirolimus: 171.8 ± 12.13 cells; p < 0.0001, Fig. 4b,c). These experiments indicate that mTORC1 mediates the beneficial effect of intranasal insulin on anesthesia-induced apoptosis in the hippocampus.

### Mice lacking mTORC1 downstream translational repressor 4E-BP2 are resistant to repeated anesthesia-induced apoptosis

**Figure 5:**
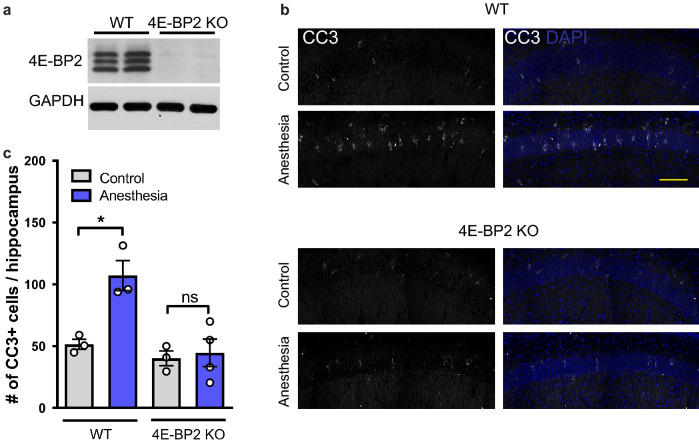
4E-BP2 knockout juvenile mice do not show apoptosis induction following repeated general anesthesia. (**a**) Western blot analysis shows the lack of 4E-BP2 protein expression in the hippocampus of 4E-BP2 knockout (KO) mice. See Supplementary Fig. 1 for full-length blots. (**b**) Representative images showing immunostaining against CC3 (white) and DAPI (blue) in hippocampal sections from wild type (WT, left) and 4E-BP2 KO (right) control and repeated anesthesia-exposed mice (1.5% isoflurane, 2 h a day at postnatal days 15–17, fixed one hour after the last anesthesia session). (**c**) Quantification of CC3-positive cells. Repeated anesthesia induces apoptosis in juvenile wild type but not 4E-BP2 knockout mice (*F*_3, 9_ = 10.13; WT control (n = 3) versus WT anesthesia (n = 3), *p < 0.05; 4E-BP2 control (n = 3) versus 4E-BP2 anesthesia (n = 4), ns (not significant), p > 0.05. Data points represent individual mice. Data are presented as mean ± SEM.

Because the inhibition of mTORC1 prevented the rescue effect of intranasal insulin on apoptosis after repeated anesthesia, we investigated if an upregulation of mTORC1 signaling could mimic the rescue effects of intranasal insulin on apoptosis using a mouse genetic approach. The major downstream effector of mTORC1 is a translational repressor, 4E-BP (Fig. 4c). Three 4E-BP isoforms are found in mammals (4E-BP1, 4E-BP2 and 4E-BP3), which are similar in function but vary in tissue distribution. 4E-BP2 is the predominant isoform found in the mouse brain^36^. Since 4E-BP2 is a repressor of translation downstream of mTORC1, the ablation of 4E-BP2 mimics the overactivation of the mTORC1/4E-BP2 signaling and allows examination of cellular effects mediated by mTORC1/4E-BP2 activation. Thus, we assessed whether juvenile *Eif4ebp2* knockout mice (4E-BP2 KO) lacking the 4E-BP2 in the brain (Fig. 5a), are resistant to anesthesia-induced apoptosis.

Wild-type (WT) and 4E-BP2 KO mice were subjected to repeated general anesthesia at PD 15–17 and apoptosis was evaluated one hour after the last anesthesia session using CC3 immunostaining. Whereas WT mice exhibited an increase in the number of CC3 positive cells in the hippocampus following repeated general anesthesia, 4E-BP2 KO mice showed no anesthesia-induced increases of apoptosis (WT control: 51.5 ± 4.07 cells; WT anesthesia: 107. 2 ± 12.18 cells, p < 0.05; 4E-BP2 KO control: 40.17 ± 5.93 cells; 4E-BP2 KO anesthesia: 44.63 ± 11.06 cells, p > 0.05, n = 3–4 mice/group, Fig. 5b,c). Collectively, these results indicate that 4E-BP2 KO mice are resistant to general anesthesia-induced apoptosis, supporting the notion that the beneficial effect of intranasal insulin is mediated via enhanced mTORC1/4E-BP2 activity.

## Discussion

In this study, we demonstrate that insulin, administered intranasally, penetrates rapidly into the brain wherein it activates mTORC1 pathway. The activation of mTORC1 mediates long-lasting neuroprotection (for at least three days after insulin administration is discontinued) against anesthesia-induced apoptosis in juvenile mice and prevents deficits in hippocampal synaptic plasticity and memory in adulthood.

### Intranasal insulin efficiently penetrates the brain to activate mTORC1

We show that intranasal insulin, labeled with FITC, reaches the hippocampus within 30 min where it activates intracellular mTORC1 signaling, as evident by increased S6 phosphorylation (Fig. 2), which is the most reliable readout of mTORC1 activity. This result is consistent with a previous report^37^, supporting the notion that intranasal insulin allows efficient delivery of insulin into the brain. The anti-apoptotic effect of intranasal insulin was prevented by the inhibition of mTORC1 selectively in the brain by icv injection of the specific mTORC1 inhibitor, temsirolimus. This indicates that the anti-apoptotic effect of intranasal insulin in the brain is mediated via its central and not systemic action.

### Activation of mTORC1 prevents apoptosis in the brain

We administered intranasal insulin to juvenile mice (PD 8–14) daily for 7 days prior to repeated exposure to general anesthesia (PD 15–17). Notably, the beneficial effects of central insulin persisted after the treatment was discontinued, indicating that insulin triggers long-lasting processes in the brain, which are protective against anesthesia-induced neurotoxicity. Since the effect of intranasal insulin on anesthesia-induced apoptosis is mediated by mTORC1, which regulates mRNA translation, it is conceivable that mTORC1-dependent changes in expression of specific proteins contribute to the long-lasting neuroprotection. Indeed, it has been shown that mTORC1 preferentially promotes translation of nucleus-encoded mitochondria-related mRNAs, enhancing mitochondrial activity and biogenesis^22,38^.

Previous studies have shown that mitochondrial dysfunction plays important roles in anesthesia-induced neurotoxicity^23,39,40^. General anesthetics increase the activity of mitochondrial complex IV and decrease the activity of superoxide dismutase (SOD) in mitochondria, resulting in an excessive production of reactive oxygen species (ROS)^23,40^, mitochondrial fragmentation and cytochrome c release from mitochondria into the cytosol^41^, which subsequently cause caspases-3 activation and apoptosis. Accordingly, reducing ROS^24^ or enhancing mitochondrial functions^24,42,43^ is sufficient to prevent anesthesia-induced neurotoxicity and behavioral deficits.

mTORC1-dependent upregulation of mitochondrial genes promotes mitochondrial functions by enhancing mitochondrial biogenesis, increasing ATP production and mitochondria fusion^22^, all processes that could contribute to resistance of cells to anesthesia-induced cellular stress. mTORC1 affects translation of mitochondria-associated genes via inhibition of 4E-BP^22,38^. 4E-BP binds and represses the activity of eIF4E, which is a cap-binding protein that stimulates translation initiation of the mammalian mRNAs by facilitating the recruitment of the ribosome to the mRNA 5′ cap^21^. Upon phosphorylation by mTORC1, the affinity of 4E-BP to eIF4E is reduced, leading to eIF4E de-repression, and stimulation of protein synthesis^44^.

The important role of mTORC1/eIF4E signaling in regulation of mitochondrial functions is consistent with our finding that 4E-BP2 KO mice are protected from anesthesia-induced apoptosis (Fig. 5). Since 4E-BP2 is a translational repressor downstream of mTORC1, 4E-BP2 deletion mimics the overactivation of mTORC1/eIF4E signaling, providing a model to study cellular effects mediated by mTORC1 stimulation. Collectively, our results indicate that the activation of mTORC1/eIF4E signaling downstream of insulin receptor is sufficient to mediate the beneficial effects of insulin in the brain. These results also support the conclusion of a previous study which proposed that the rescue effect of intranasal insulin in adult mice is mediated via the activation of mTORC1 pathway^45^.

Interestingly, a previous study using P magnetic resonance spectroscopy (P-MRS) imaging showed that intranasal insulin in humans increases brain energy metabolism^46^, as individuals receiving intranasal insulin had higher levels of ATP and phosphocreatine in the brain. It is therefore conceivable that intranasal insulin protects neurons from apoptosis by promoting mTORC1-dependent increases in mitochondrial functions, energy production, and potentially stimulating other anabolic process (lipid biogenesis, inhibition of autophagy), enhancing the capacity of brain cells to cope with anesthesia-induced stress and neurotoxicity during and after anesthesia.

### Intranasal insulin rescues deficits in synaptic plasticity and memory formation

We show that pre-treatment of juvenile mice with intranasal insulin rescues deficits in both synaptic plasticity (L-LTP) and hippocampus-dependent memory in adult animals. Because L-LTP is a putative mechanism of learning and memory, rescue of hippocampal L-LTP and memory by intranasal insulin is consistent with the idea that memory deficits are caused by impaired synaptic plasticity. The long-lasting impairment in synaptic plasticity could be due to a repeated anesthesia-induced loss of key cellular subpopulations which are critical for the normal functioning of neuronal circuits underlying learning and memory. It is therefore essential to decipher if there are specific cell type(s) that are more affected and undergo preferential apoptosis following repeated anesthesia.

Our study shows that deleting 4E-BP2 is protective against apoptosis in vivo. It is important to investigate if neurons in 4E-BP2 KO mice are also protected from apoptosis in other models of cellular stress such as epilepsy or neurodegenerative disorders. Since the 4E-BP2 KO mice have been shown to exhibit deficits in L-LTP and hippocampus-dependent memory formation^45^, we were unable to study if these mice are also protected from anesthesia-induced impairments in synaptic plasticity and memory.

Our study has several limitations. We focused on apoptosis in hippocampus and hippocampal-dependent memory formation. However, anesthesia-induced apoptosis has been also observed in other brain areas, such as cortex and thalamus^1–3,47^. Therefore, future work should be undertaken to study the effect of intranasal insulin on anesthesia-induced deficits in other brain areas. Our results show that a combination of anesthesia with intranasal insulin and temsirolimus induces significantly greater apoptosis than anesthesia alone (Fig. 4c). It remains unclear why temsirolimus exacerbates apoptosis in combination with intranasal insulin and future studies should clarify this point.

Our data strongly indicate that intranasal insulin acts via mTORC1/4E-BP2-dependent mechanisms, which are known to promote mitochondrial functions and other anabolic processes. However, intranasal insulin could also alleviate neurotoxicity via modulation of brain inflammatory responses. Insulin has anti-inflammatory actions and intranasal insulin improves cognitive functions and decreases neuroinflammation following traumatic brain injury in rats^48^.

In our study, repeated anesthesia was performed on 15–17 day-old mice, corresponding to ~ 2-year-old children^26^, although the majority of research examining the effect of general anesthesia in juveniles is predominantly performed on postnatal day 7 mice and rats^49^, which corresponds to a late-term fetus or neonate (< 3 months of age). The later age for postnatal anesthesia was selected because most surgeries in children occur when they are approximately 1–3 years of age^25,50^, which is equivalent to a ~ PD 15 mouse^26^. Additionally, control of hemodynamic functions in younger mice is challenging.

Despite the compelling epidemiological evidence in humans linking repeated exposure to general anesthesia in young children to learning disabilities and attention-deficit hyperactivity disorder (ADHD), effective treatments are lacking. Our clinical trial on intraoperative normoglycemia showed that high-dose insulin lasting 4 h, adequate time for insulin to cross the blood–brain barrier (BBB), preserved verbal learning memory 2 and 7 months after cardiac surgery^51^. Unfortunately, traditional methods of insulin administration (intravenous or subcutaneous) cause a pronounce drop in blood glucose levels and require judicious monitoring. This side effect is avoided by intranasal administration. Intranasally administered insulin, though off-label, has been utilized in rodents drop-wise to the nares and in humans via a meter-dosed nasal spray^12,13,52,53^, showing promising results. This simple non-invasive delivery method into the central nervous system circumvents severe hypoglycemia with systemic insulin administration^52^ and provides a readily available and low-cost approach to deliver insulin to the brain to prevent repeated anesthesia-induced neurotoxicity and long-lasting cognitive deficits.

In summary, our work advances the understanding of the link between repeated general anesthesia-induced apoptosis, and deficits in synaptic plasticity and memory. Moreover, it uncovers the role of insulin and mTORC1/eIF4E signaling in preventing apoptosis, and offers a potential therapeutic approach for this emerging clinical problem.

## Materials and methods

### Animals and environment

C57BL6 mice were bred in-house at McGill University animal facility. *eIF4ebp2* knockout mice (on pure C57BL6 background) and their corresponding wild type controls were provided by Dr. Sonenberg (McGill University). Behavioural experiments were performed on male and female mice, however, no sex-specific differences were noted. The subsequent experiments were performed on male mice. Food and water were provided ad libitum, and mice were kept on a 12:12 h light/dark cycle (lights on at 07:00 h).

All procedures complied with Canadian Council on Animal Care guidelines and were approved by the McGill University Facility Animal Care committee (FACC). This study was carried out in compliance with the ARRIVE guidelines^54^. The experimenter was “blind” to the genotype/condition in all studies.

### Anesthesia and intranasal insulin administration

Repeated general anesthesia was performed during postnatal days 15–17 (3 days in total) for 2 h a day using 1.5% isoflurane (Aerrane, Baxter, Canada, in 100% oxygen), comparable to pediatric MAC of 1.8%. The airflow rate was 2 L/min. The animals were anesthetized in a bottom-heated chamber (8 inches × 4 inches × 5 inches) and protective eye gel (Systane ointment) was applied at the beginning of each anesthesia session. Animals were turned over every 30 min. Breathing pattern was monitored every 10 min and the depth of anesthesia was adjusted accordingly. At the end of the anesthesia session, mice were allowed to recover in a bottom-heated cage.

Mice were given 20 µl daily intranasal insulin (Humulin R, 100 units/ml, Eli Lilly, Toronto, Canada) or isotonic saline (0.9% sodium chloride injection) at postnatal days 8 -14. The protocol was modified from adult^14,28,55^ and juvenile intranasal insulin delivery^29,30^. Intranasal administration was performed manually without anesthesia by holding the pup in a supine position and placing a 10 μL drop of the solution to cover the opening of both nostrils, and not forcibly into the nares. The mice were held supine for 5 s, allowing the mouse to inhale a volume suitable to their size. The mice were allowed to recover for 1 min before repeating the procedure.

Temsirolimus (PZ0020, Sigma-Aldrich, USA) was applied via freehand icv injection (2 µl at 50 µg/µl) as described previously^56,57^. Briefly, a Hamilton 1701RN 10 µl syringe with a 2-inch 26-gauge needle was fitted with a stiff tubing to expose 2.5 mm of the needle, from bevel tip, to standardize injection depth. Mice were anesthetized with 2% isoflurane for 2–3 min and placed on a sternal recumbent position. Bregma was identified by drawing an imaginary point midline between the anterior base of the ears and feeling for the suture with the needle tip. The needle was inserted at a 45-degree angle, bevel up, 2 mm below and 2 mm lateral to bregma. 2 µl of the solution was injected slowly to each ventricle and the needle held in place for 30 s to prevent backflow of the drug. Standard aseptic technique was used for the icv injections.

### Novel object location

All experiments were performed by a researcher blind to experimental condition. Object location memory was run over 5 days. Day one comprised of two 1-min sessions of handling the animals (morning and afternoon). Day two consisted of a morning handling session and a 10-min afternoon habituation session in the empty arena (arena dimensions: 60 cm × 60 cm × 30 cm). Two 10-min training sessions (separated by 4 h) with similar objects placed in the middle between the corners close to the wall (opposite walls for two objects) occurred on day three and day four (total of 4 training sessions). Testing (10 min) was performed in the morning of day five where one object was randomly moved to a corner of the arena. The mice were recorded using a video camera. Discrimination index was calculated as time spent exploring the moved object minus time exploring unmoved object over the total time exploring both objects.

### Contextual fear conditioning

Contextual fear conditioning was performed on the mice which underwent novel object location test. Training protocol consisted of a 2-min period of context exploration, followed by two 1 s 0.6 mA foot shocks separated by 60 s. The mice were returned to their home cage 1 min after the second foot shock. The mice were tested for contextual fear memory by placing them 24 h later in the training chamber for 3 min. The incidence of freezing was scored in 5-s intervals as either “freezing” or “not freezing”. Percent of freezing indicates the number of intervals in which freezing was observed divided by total number of 5-s intervals.

### Field potential recordings

Male mice (8-week-old) were anesthetized with isoflurane and the brain was rapidly removed and placed in ice-cold oxygenated artificial cerebrospinal fluid (ACSF) containing 124 mM NaCl, 2.5 mM KCl, 1.25 mM NaH_2_PO_4_, 1.3 mM MgSO_4_, 2.5 mM CaCl_2_, 26 mM NaHCO_3_ and 10 mM glucose. Transverse hippocampal slices (400 μm), prepared using Leica VT1200S Vibratome, were allowed to recover submerged for at least 2 h at 32 °C in oxygenated ACSF and for additional 30 min in a recording chamber at 27–28 °C while perfused with ACSF.

Field extracellular postsynaptic potentials (fEPSPs) were recorded in CA1 stratum radiatum with glass electrodes (2–3 MΩ) filled with ACSF. Schaffer collateral fEPSPs were evoked by stimulation with a concentric bipolar tungsten stimulating electrode placed in mid-stratum radiatum proximal to CA3 region. Baseline stimulation was applied at 0.033 Hz by delivering 0.1 ms pulses, with intensity adjusted to evoke 35% of maximal fEPSPs. Theta-burst stimulation (TBS) consisted of fifteen bursts of four pulses at 100 Hz separated by 200 ms intervals. The experiments were performed by researcher blind to the experimental condition.

### Immunohistochemistry and image analysis

Mice were sacrificed one hour after the last anesthesia session (postnatal day 17) by intracardiac perfusion with 4% paraformaldehyde. The brains were post-fixed at 4 °C and 50 µm-thick sequential coronal sections were taken from the beginning to the end of the dorsal hippocampus using a Leica VT1200S vibratome. Three sequential hippocampal sections, 200 µm apart, from both dorsal hippocampi per mouse were used for immunohistochemistry.

Sections were washed three times for 5 mins with 0.3% Triton X-100 in PBS, in a shaker. Sections were permeabilized and blocked with 10% goat serum in PBS Triton-X 0.2% (PBST) for 1 h, then, incubated with the primary CC3 antibody (1:100 in PBST, STJ 97448) at 4 °C overnight, washed three times in PBS and incubated with the secondary antibody (Alexa Fluor 488, 1:500 in PBST) for one hour. Sections were washed once with PBST, then PBS alone, and lastly with PBS and DAPI at a 1:5000 dilution in PBS.

The sections were mounted on microscope coverslips using Invitrogen ProLong Gold antifade reagent (Thermo Fischer Scientific) and images were acquired with a Zeiss epi-fluorescence microscope equipped with Apotome using a 20 × objective. The number of CC3-positive cells per section was counted and averaged between both dorsal hippocampi (left and right, all hippocampal areas). Three sections per mouse were analyzed for CC3-positive cells by a researcher blinded to the experimental condition or genotype, and the sum of CC3-positive cells in three sections was calculated and presented. Image processing was performed with ImageJ (NIH).

### Western blotting

Tissue extraction for Western blotting was prepared in ice-cold RIPA buffer (Cat. No. R0278, Sigma-Aldrich) and included protease inhibitor cocktail (Cat. No. 4693132001, Sigma-Aldrich) and phosphatase inhibitor cocktail (cocktail 2, 1:100, Cat. No. P5726-1ML, Sigma-Aldrich and cocktail 3, 1:100, Cat. No. P0044-1ML, Sigma-Aldrich). Following centrifugation at 10,000×*g* for 10 min, the supernatant protein concentration was measured. Equal protein quantities were boiled for 5 min in sample buffer and separated by SDS-PAGE. Following electrophoresis, proteins were transferred to 0.2 mm nitrocellulose membranes. Membranes were blocked in 5% BSA in Tris-buffered saline containing 0.1% Tween-20 (TBS-T) for 1 h prior to overnight incubation with primary 4E-BP2 (1:1000, Cat#2845, Cell Signaling Technology) and β-actin (1:5000, Cat#A5441, Sigma) antibodies. The membranes were then washed, incubated for 1 h with HRP-conjugated secondary antibody, washed again, treated with Enhanced Chemiluminesce reagent (Perkin Elmer) and exposed to autoradiography films (Denville Scientific Inc.).

### Statistical analyses

All results are expressed as mean ± SEM. All statistical tests were made using a one-way ANOVA followed by between-group comparisons using Tukey’s post hoc test, with p < 0.05 as significance criteria (GraphPad Prism 7.03), except Fig. 1g, Fig. 1k, and Fig. 4a where a student’s t-test was used, *p < 0.05, **p < 0.01, ***p < 0.001 and ****p < 0.0001.

## Supplementary Information

Supplementary Legend.

Supplementary Figure 1.
