# Supplementary material for: Intranasal insulin rescues repeated anesthesia-induced deficits in synaptic plasticity and memory and prevents apoptosis in neonatal mice via mTORC1
Source: Sci Rep. 2021 Jul 29;11:15490. doi: 10.1038/s41598-021-94849-3 (PMC8322102; doi:10.1038/s41598-021-94849-3)
Supplement: Supplementary file 1 — Supplementary Legend. [file 41598_2021_94849_MOESM1_ESM.docx]

**Supplementary figure 1. Lack of 4E-BP2 in 4E-BP2 knockout animals.** Hippocampi from wild-type (WT) and 4E-BP2 knockout (KO) mice were extracted from 8-week-old male mice and subjected to western blot analysis. Top blot shows that 4E-BP2 KO animals lack 4E-BP2 protein. Rectangle outlines the area shown in Fig. 5a. Bottom blot shows GAPDH that was used as a loading control. Rectangle outlines the area shown in Fig. 5a.
