# Supplementary figures and images for: Intranasal insulin rescues repeated anesthesia-induced deficits in synaptic plasticity and memory and prevents apoptosis in neonatal mice via mTORC1
Source: Sci Rep. 2021 Jul 29;11:15490. doi: 10.1038/s41598-021-94849-3 (PMC8322102; doi:10.1038/s41598-021-94849-3)

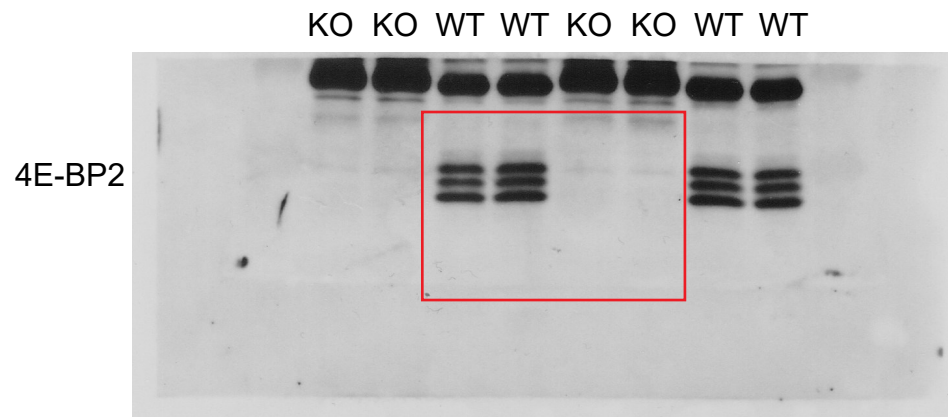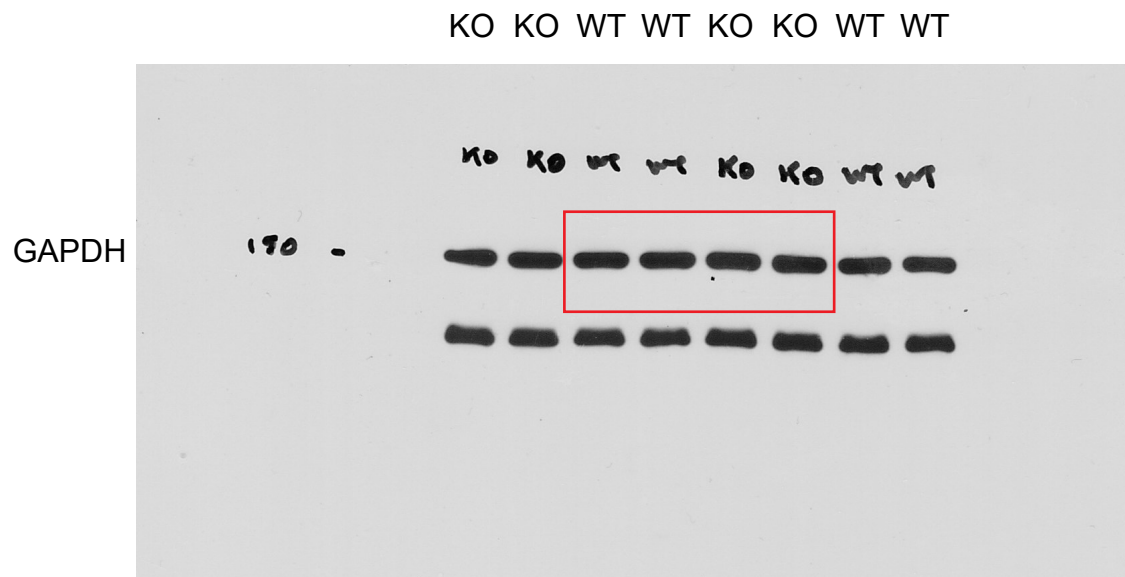

Supplement: Supplementary file 2 — Supplementary Figure 1. [file 41598_2021_94849_MOESM2_ESM.pdf]
